# CO-OCCURRENCE OF ABUSE AND NEGLECT AS REPORTED BY DEMENTIA FAMILY CAREGIVERS

**DOI:** 10.1093/geroni/igz038.2002

**Published:** 2019-11-08

**Authors:** Carolyn E Pickering, Maria Yefimova, Christopher Maxwell

**Affiliations:** 1 University of Texas Health Science Center San Antonio, San Antonio, Texas, United States; 2 Stanford Healthcare & VA Palo Alto, Menlo Park, California, United States; 3 Michigan State University, East Lansing, Michigan, United States

## Abstract

Understanding the co-occurrence or overlap among multiple forms of elder abuse and neglect (EAN) is important for designing effective interventions. This paper reports patterns of family caregiver’s daily behaviors related to physical assault, psychological mistreatment, and neglect. Majority of participants self-reported at least one EAN behavior (74%), with most reporting using multiple forms of EAN over the 21-day period (52%). On a given day, psychological mistreatment and neglect were more likely to happen in isolation, while physical assault was more likely to co-occur with psychological mistreatment. The mixed model’s intra-class coefficient suggests the daily context, rather than personal characteristics, explain the variance in the use of EAN. These findings highlight the importance of never minimizing a single event of EAN reported in clinical practice, give the high rate of polyvictimization, and reinforces the need to understand why caregivers use one form of EAN over another on a given day.

